# Maximizing ball movement unpredictability in association football: A Rényi entropy-based approach to optimizing event distribution randomness

**DOI:** 10.1371/journal.pone.0326800

**Published:** 2026-02-25

**Authors:** Ishara Bandara, Sergiy Shelyag, Sutharshan Rajasegarar, Dan Dwyer, Eun-jin Kim, Maia Angelova

**Affiliations:** 1 School of IT, Deakin University, Melbourne, Victoria, Australia; 2 Research Centre for Fluid and Complex Systems, Coventry University, Coventry, United Kingdom; 3 College of Science and Engineering, Flinders University, Adelaide, South Australia, Australia; 4 Centre for Sport Research, Deakin University, Melbourne, Victoria, Australia; 5 Aston Digital Futures Institute, Aston University, Birmingham, United Kingdom; 6 Institute for Biophysics and Bioengineering, Bulgarian Academy of Sciences, Sofia, Bulgaria; Portugal Football School, Portuguese Football Federation, PORTUGAL

## Abstract

Modern football prioritizes team play and tactical strategies over individual brilliance. However, its low-scoring nature makes evaluating team performance challenging. Unpredictable ball movement enhances offensive play while complicating defensive setups. To better capture this dynamic nature, authors’ prior work has proposed entropy-based time-series metric to assess unpredictable ball movement by quantifying Spatial Event Distribution Randomness (EDRan). However, some teams may prefer to dominate specific areas with unpredictability, while others utilize the entire field. Existing literature has not examined whether emphasizing dominant (frequently used field regions for ball movement) or considering all regions equally, including rarely used areas, is a more effective approach for computing randomness in event distribution. Moreover, existing research has not investigated the underlying patterns of event distribution randomness, particularly how these variations differ between winning and losing teams, both in terms of overall field coverage and concentration within dominant regions. This study addresses these gaps by analyzing event distribution randomness using Rényi entropy with varying alpha values (0≤α≤20).Correlation analysis indicated that assigning equal weight to all field regions, including rarely used areas, with Max entropy (α=0alpha) was most strongly associated with match-winning performance. In men’s data, machine learning models trained with α=0,0.1,alpha and 0.5 achieved statistically significant improvements over models trained with the traditionally used Shannon entropy (α→1alpha). These results suggest that unpredictability distributed across the entire field, maximizing the use of diverse regions, is more strongly associated with success than randomness restricted to dominant areas. The best-performing model, obtained with α=0alpha, significantly outperformed both the baseline and existing models in the literature, achieving an accuracy of 80.61% in predicting match winners.

## 1 Introduction

Association football is a highly dynamic and strategic sport where team coordination and tactical execution play a crucial role in a team’s success. Thus, the team’s manager or tactical decision maker’s role is highly valued by most teams rather than individual star players. Unlike sports with frequent scoring ways and opportunities, association football is a low scoring invasion sport where the team attacks while defending attacks from opposition and shooting at goal is the only way of scoring [1,2]. Association football can be viewed as a complex system [3], and its results are often influenced by chance or luck [4–6]. Thus, performance evaluation becomes challenging, often requiring advanced analytical approaches to evaluate team performance.

This unpredictability and randomness in match outcomes can be influenced by controllable factors such as tactics [7] and skill [8], as well as uncontrollable factors like chance or luck [4,5]. Since scoring in association football is only possible through shots on goal, widely used offensive performance metrics, such as expected goals (xG), assess the probability of scoring based on statistical factors influencing shot success, including distance from goal, shooting angle, and goalkeeper positioning [9–11]. However, recent studies have introduced expected goals models that account for the sequence of events leading to a shot, demonstrating that prior events significantly influence shot success probability [12].

Ball possession is another commonly analyzed performance metric in the literature [13–16]. While some studies suggest a positive correlation between possession and match-winning performance [13,14], others report no significant relationship between possession and victory probability [15,16]. These conflicting findings may be due to lack of time-series analysis, as teams adjust their strategies based on various factors such as game state and opposition strength [17,18]. Guan et al. (2023) revealed that teams leading by a single goal adopt a more cautious approach, particularly in the latter stages of a match or when facing a strong opponent or when leading by only one goal [17,18]. Additionally, some teams prioritize possession-based tactics, emphasizing ball retention, whereas others favor direct play, focusing on long passes rather than prolonged possession. Existing research indicates that “direct-play” strategies and counterattacks are more effective than possession-based tactics [7,19]. For these reasons, possession alone may not provide comprehensive insights into team performance.

Other traditional methods for evaluating offensive performance have often relied on event-based statistics, such as the number of shots, shots on target, and completed passes [20–23]. However, these event-based metrics fail to capture the temporal aspects of the game and often overlook the complexity of ball movements and structured or unstructured tactical plays.

Open space in the field available for the ball-carrier has been evaluated in the literature as an alternative performance evaluation metric to evaluate offensive performance as today’s football tactics focus on creation of open space to create scoring opportunities [24–27]. However, in order to create open spaces in the field, opposition defense should be disrupted. Therefore, both offense and defense play a crucial role in invasion sports such as association football. In defense, swift decision making is necessary to stop opposition attacks. However, predictable ball movements by the offensive team can make it easier for opposition defenders to defend the attacks. Recent research has explored entropy-based models to quantify randomness in ball movements, considering spatial event distribution randomness (EDRan) [28] and player-to-player interactions [29–31] offering a new perspective on offensive unpredictability.

While some of the previous work has evaluated the player to player interaction randomness as a performance evaluation metric [29–31], players subject to substitutions, role changes, and positional adjustments. Therefore, as a solution, Spatial Event Distribution Randomness (EDRan) has been proposed by the authors, as an alternative measure to quantify randomness in team performance assuming that ball movement is more critical than player movement for analysis of offensive team performance [28]. EDRan has been introduced as a time-series team performance evaluation metric that exhibits a positive correlation with match-winning performance. In previous studies, a simple machine learning model developed with Generalized Linear Model (GLM), utilizing only EDRan data collected across ten time intervals achieved an accuracy of 79.95%, highlighting its significance as a team performance evaluation metric in association football. For quantifying spatial event distribution randomness (EDRan), previous research proposed segmenting the football field into predefined regions and generating probability distributions based on ball movement events within each region. Shannon entropy, which accounts for each region proportionally to its ball movement events, was used as the metric to measure randomness in these distributions.

However, football tactics vary from team to team. Modern football tactics continue to evolve [32], with some teams emphasizing possession-based play to control the ball and create goal-scoring opportunities, while others adopt “direct-play” or defensive strategies, allowing the opposition to attack and capitalizing on counterattacks. Additionally, while some teams prefer certain regions of the field (e.g, some teams prefer flanks more while some teams prefer central channel to move the ball), others utilize the full width of the field to create space. As a result, certain teams may dominate specific areas, while some may try to distribute their actions more randomly across the field [33,34]. Taki et al. defined a player’s dominant region as the area they can reach before any other player and when the dominant regions of all players on a team are combined, this forms the team’s dominant region [33]. However, earlier studies have not evaluated whether assigning greater or lesser emphasis on dominant or rarely used regions are effective for quantifying EDRan and has not explored how the temporal variation in these regions differs between men’s and women’s games.

To address these gaps in the literature, this study investigates whether placing greater emphasis on more frequently used regions, assigning equal weight to all regions, including rare ones, or utilizing Shannon entropy, which proportionally weights all regions based on their probabilities, correlates more strongly with match-winning performance. As an alternative to Shannon entropy, this study employs Rényi entropy with varying *α* values (0≤α≤2) to determine whether an entropy measure that is more sensitive to rare events or one that prioritizes frequently used regions is more suitable for quantifying event distribution randomness (EDRan). This study defined dominant and rare regions according to how frequently they were used for ball movement.

- Dominant regions are defined as areas of the field that teams frequently utilize for ball movement, where the likelihood of a ball movement event (e.g.: pass) occurring is significantly higher compared to other regions.
- Rare regions are defined as areas of the pitch that teams infrequently utilize for ball movement, where the likelihood of a ball movement event occurring is significantly lower compared to other regions.

Rényi entropy, introduced by Alfred Rényi in 1961 [35], generalizes classical Shannon entropy [36] by incorporating a parameter *α*, providing a more flexible framework for quantifying information diversity.

The analysis of this work was conducted separately for men’s and women’s game data, focusing on the temporal dynamics of EDRan computed using Rényi entropy across varying *α* values. Match-winner prediction models were developed using data across several *α* values, and based on these results, an advanced model was proposed to investigate the relationship between temporal performance metrics and match outcomes.

Investigating these aspects could contribute to improve the accuracy of match outcome prediction models based on EDRan, facilitating more effective assessments of team performance that are independent of final scores. Moreover, it facilitates deeper insights into gameplay dynamics, including temporal patterns, the extent to which, randomness in dominant versus less utilized rarer regions correlates with winning outcomes, and how such randomness evolves in relation to time.

## 2 Materials and methods

Ethics approval for this study was obtained from the Coventry University Ethics Approval Committee (Approval Number: P174511). All procedures performed in this study were in accordance with the ethical standards of Coventry University, UK.

This study employed a retrospective observational design based on secondary data from professional football matches. A cross-sectional comparative framework was applied, analyzing event distribution randomness across matches to compare winning versus losing teams. No interventions were introduced, and all analyses were conducted on previously recorded match event data.

**Fig 1:**
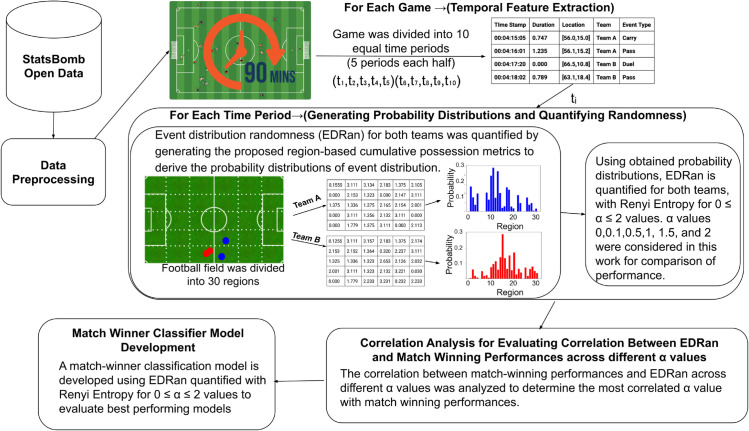
Summarized framework of this study.

Considering that some teams concentrate ball movement in some specific regions of the field (dominant regions) while others distribute play across the entire field, this study compares three approaches to quantifying spatial event distribution randomness (EDRan): (i) emphasizing dominant regions (frequently used regions for ball movement), (ii) assigning equal weight to all regions, including rarely used ones, and (iii) using Shannon entropy, which weights regions proportionally to their event distribution probabilities. To facilitate this comparison, Rényi entropy is applied with *α* values in the range 0≤α≤2, using region-based cumulative possession matrices as proposed in past investigations [28]. Football field is divided into 30 equal area named regions, each game is divided into ten time intervals, five equal time intervals per half (*t*_*i*_;1≤i≤10), and region-based cumulative possession matrices are constructed to derive the probability distribution associated with a team’s event distribution during the considered *t*_*i*_ time period. These probability distributions are then used to quantify randomness with different *α* values in Rényi entropy, allowing for the identification of the optimal *α* value for EDRan through a correlation analysis with match-winning outcomes and development of match winner prediction models. Fig 1 provides an overview of the summarized framework of this study.

All data processing and analyses in this work were conducted in Python 3.11.13 using scikit-learn 1.6.1, pandas 2.2.2, numpy 2.0.2, matplotlib 3.10.0, and scipy 1.16.1 within the Google Colab environment. The experiments in this study were executed on commodity hardware in a CPU-only environment, using a personal laptop (16 GB RAM); no GPUs were employed.

### 2.1 Data

This work used the StatsBomb open data set [37], which is a publicly available event-log dataset containing match data from top-tier men’s and women’s international and club competitions in Europe. Given the technical and physiological differences between men’s and women’s football in existing literature [38], men’s and women’s football games were evaluated separately.

The dataset included detailed event-log data, such as event location, event type, ball possession team, event duration, and player involvement. Event locations were mapped onto a 120×80 pixel grid, oriented in the direction of the possession team’s attack, with the *x*-coordinate representing the field’s length and the *y*-coordinate representing its width. In the StatsBomb dataset, teams are labeled as *Home team* and *Away team*. However, these labels do not necessarily reflect the actual home or away status of the teams, they are used solely as a naming convention. To avoid confusion, this work instead refers to the two sides as “Team A” and “Team B”, which correspond respectively to the *Home team* and *Away team* designations in the StatsBomb dataset. Men’s dataset consisted of games from 116 men’s teams while women’s dataset consisted of games from 54 women’s teams.

Games without a match winner were excluded from the dataset, as the study aims to assess the temporal contributions of the considered temporal metrics to match-winning performances. Additionally, to maintain consistency in temporal feature extraction, where each game is divided into ten equal time intervals, only matches that concluded with a winner within regular playing time (two halves) were considered. Remaining men’s dataset consisted of 608 games where “Team A” has won 342 games (56%) and “Team B” has won 266 games (44%). Remaining women’s dataset consisted of 374 games where 206 games (55%) has been won by “Team A” and 168 games (45%) has been won by “Team B”.

To assess adequacy of sample size, a post hoc power analysis was conducted for a two-sided one-sample proportion test against *p*_0_ = 0.50 at α=0.05. For the men’s dataset (*n* = 608, 342/608 = 0.5625), the estimated power was 1−β≈0.87, indicating sufficient power to detect the observed deviation from 50–50; for the women’s dataset (*n* = 374, 206/374 = 0.5508), the power was 1−β≈0.502, indicating the sample is underpowered. This limitation is acknowledged, and while analyses are reported for both datasets, no definitive conclusions are drawn from the underpowered women’s dataset.

### 2.2 Event distribution randomness

A previous study introduced event distribution randomness (EDRan) as a time-series team performance evaluation metric for assessing unpredictable ball movement performance in association football [28]. This approach involves dividing the field into 30 equal segments and constructing region-based cumulative possession matrices for each team over a defined time period to estimate the probability distribution of spatial event distributions.

To generate “Region-based Cumulative Possession Matrices”, the football field is divided into 30 equal-area regions, as proposed in [28]. Liu et al. (2016) proposed dividing the association football field into thirty regions to analyze passing patterns using data mining techniques [39]. In the authors’ previous work [28], this method was refined by dividing the field into 30 equal-area regions, as unequal region sizes could bias the measurement of randomness. The present work adopts the same equal-area division of the field into 30 regions. This is achieved by subdividing each third of the field (attacking third, central third, and defensive third), as defined in the literature [40,41], into 10 equal regions. However, different field partitioning schemes were also evaluated, including increasing the number of regions (e.g., 96 regions (12 columns × 8 rows)) and reducing it (e.g., 15 regions (5 rows × 3 columns)). The analysis indicated that dividing the field into a higher number of regions resulted in a sparse distribution of events, with many regions containing very few or no events, which consequently led to poor model performance. In contrast, reducing the number of regions resulted in all teams utilizing every region during play, thereby limiting the discriminatory power of the randomness analysis. The 30-region configuration offered the best balance, yielding more informative distributions.

**Fig 2:**
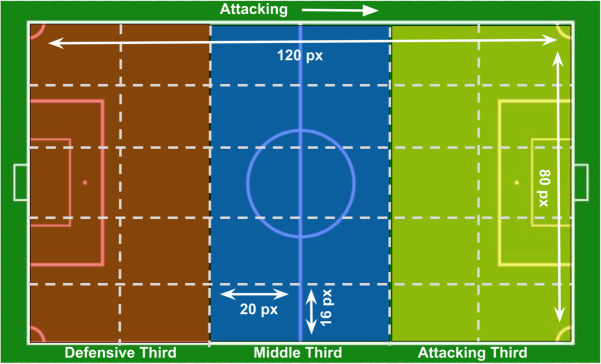
Division of football field into 30 regions.

This approach enables the computation of event distribution in terms of ball movement, independent of individual players, as players are subject to substitutions, role changes, and positional adjustments. Thus, it is assumed that ball movement is more critical than the player movement for analysis of team performance. Fig 2 shows the division of football field into 30 regions.

In this study, each game is divided into ten equal time intervals $(t_{i}{)}_{i=1}^{10}$, with $t_{1\ldots 5}$ covering the first half and $t_{6\ldots 10}$ the second. Unlike [28], which segmented the full match into ten equal parts without respecting the half-time break, allowing the fifth interval to contain events from both halves due to added (injury) time at the end of each half. The present approach first identifies the half-time boundary and then divides each half (including its injury time) into five equal intervals. This prevents cross-half mixing and yields half-specific, temporally aligned segments that are comparable across matches. In association football, the halftime break is followed by approximately 45 minutes of play, allowing players to rest, switch sides, and revise tactical strategies. The previous approach often placed both the end of the first half and the beginning of the second half within the same interval typically the fifth, potentially ignoring important temporal patterns. By segmenting each half separately, this study preserves the distinct dynamics at the end of the first half and the beginning of the second half, ensuring more accurate temporal modeling. However, both increasing and decreasing the number of time periods were also examined. A reduction in the number of periods resulted in a high concentration of events within each time period and a diminished set of temporal features. In contrast, increasing the number of periods produced fewer events per time period, leading to a greater number of sparse regions. To achieve a balanced configuration, 10 time periods (5 for each half) were employed.

**Fig 3:**
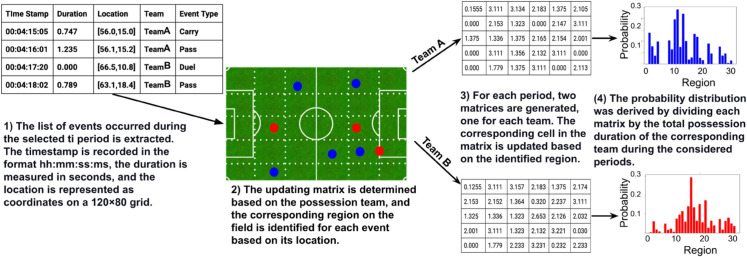
The steps of generating region-based cumulative possession matrices to compute probability distributions of event distributions, as outlined in [28].

Within each interval, a region-based cumulative possession matrix is constructed for both teams by analyzing ball-movement events occurring during that period. This matrix, structured as a 5×6 matrix (30 values), represents 30 distinct field regions. For each ball-movement event, the corresponding team’s matrix is updated by adding the event duration to the respective cell associated with the field region where the event occurred. Off-ball events were not included in this study due to dataset limitations and because their analysis lies beyond the scope of the present work, which focuses on examining randomness in ball movement and its association with match-winning performance. By iterating through all events within a given time interval, the resulting matrix captures the total ball-carrier event duration for each region. To derive the probability distribution of event distributions, the matrix values are normalized by dividing the matrix by the team’s total event duration within that interval. This process is repeated for both teams across all ten time intervals in each match. Fig 3 shows the steps of generating region-based cumulative possession matrix to derive probability distributions of event distributions as proposed in [28].

Subsequently, an entropy measure is applied to quantify event distribution randomness using the generated probability distributions for both teams in each game across the 10 defined time intervals.

In earlier work, Shannon entropy was proposed as a measure of calculation of entropy using the probability distribution generated by the region-based cumulative possession matrix [28]. Shannon entropy is a measure of uncertainty in information theory, quantifying the randomness of a probability distribution [36]. A higher Shannon entropy value indicates greater randomness, whereas a lower entropy value suggests a more predictable distribution.

However, Shannon entropy assigns proportional importance to all 30 regions based on their event distribution probabilities. This raises the question of its suitability and leaves space for improvement, as some football tactics focus on dominating specific field regions (by frequently using specific regions of the field for ball movement (e.g., flanks)), while some emphasize utilizing the entire playing area for ball movement, increasing spatial dominance. To address this, this study evaluates the quantitative value of EDRan with Rényi entropy with 0≤α≤2 to determine which value of *α* serves as a more appropriate entropy measure. Rényi entropy generalizes this concept by allowing flexibility to emphasize either dominant or rare events, making it more suitable for capturing tactical nuances such as reliance on specific passing patterns in football.

Rényi entropy is a generalized measure of entropy that introduces a parameter *α* to control the weighting of probabilities, providing a flexible way to quantify randomness. It is defined as:

$$H_{\alpha }(X)=\frac{1}{1-\alpha }\log\sum_{i=1}^{n}p_{i}^{\alpha }$$ (1)

where,

*p*_*i*_ represents the probability of event *i*,

*n* is the number of possible states

*X* is the discrete random variable

*α* is the order of entropy (where 0≤α and α≠1)

When α=0, Rényi entropy simplifies to the Max entropy (or Hartley entropy):

Substituting α=0 to Rényi entropy


$$H_{0}(X)=\frac{1}{1-0}\log\sum_{i=1}^{n}p_{i}^{0}.$$

Since any nonzero probability raised to the power of zero equals one, the summation simplifies to the number of nonzero probability terms, denoted as *N*:


$$\sum_{i=1}^{n}p_{i}^{0}=N.$$

Thus, the expression for *H*_0_(*P*) simplifies to:


$$H_{0}(X)=\log N.$$

This corresponds to Max entropy, which quantifies the logarithm of the number of nonzero probability states.

$$H_{0}(X)=\log|\{i|p_{i}>0\}|$$ (2)

When α→1, Rényi entropy simplifies to the Shannon entropy:


$$H_{\alpha }(X)=\lim_{\alpha \to 1}H_{\alpha }(P).$$

Substituting the definition of Rényi entropy:


$$H_{\alpha }(X)=\lim_{\alpha \to 1}\frac{1}{1-\alpha }\log\sum_{i=1}^{n}p_{i}^{\alpha }.$$

To evaluate this limit, L’Hôpital’s rule is applied:


$$H_{\alpha }(X)=\lim_{\alpha \to 1}\frac{\frac{d}{d\alpha }\log\sum_{i=1}^{n}p_{i}^{\alpha }}{\frac{d}{d\alpha }(1-\alpha )}.$$

Taking the derivatives:


$$H_{\alpha }(X)=\lim_{\alpha \to 1}\frac{\frac{\sum_{i=1}^{n}p_{i}^{\alpha }\ln p_{i}}{\sum_{i=1}^{n}p_{i}^{\alpha }}}{-1}.$$

$$H_{\alpha }(X)=\lim_{\alpha \to 1}-\frac{\sum_{i=1}^{n}p_{i}^{\alpha }\ln p_{i}}{\sum_{i=1}^{n}p_{i}^{\alpha }}.$$

Since $p_{i}^{\alpha }\to p_{i}$ as α→1, the final expression simplifies to:

$$H_{1}(X)=-\sum_{i=1}^{n}p_{i}\log p_{i}.$$ (3)

This result corresponds to Shannon entropy.

When α=2, Rényi entropy simplifies to the collision entropy:

Substitute α=2 to Rényi entropy:


$$H_{2}(X)=\frac{1}{1-2}\log\sum_{i=1}^{n}p_{i}^{2}$$

$$H_{2}(X)=-\log\sum_{i=1}^{n}p_{i}^{2}$$ (4)

- For α<1, EDRan computed with Rényi entropy is more sensitive to rare events.
- For α>1, EDRan computed with Rényi entropy becomes less sensitive to rare events and places greater emphasis on dominant events.

The event distribution randomness was quantified using Rényi entropy with *α* values at intervals of 0.5 ranging from 0 to 2. Specifically, α=0 (Max Entropy), 0.5, 1 (Shannon Entropy), 1.5, and 2 (Collision Entropy) for both teams in each game, across 10 time periods. Additionally, to evaluate how the distribution behaves as *α* approaches zero, α=0.1 was also included in the analysis.

### 2.3 Data preprocessing

The event distribution randomness (EDRan) of ball movement was extracted for both teams across each time interval *t*_*i*_ in every game, for both the men’s and women’s datasets. Event distribution randomness was computed using Rényi entropy with *α* values of 0 (Max entropy), 0.1, 0.5, 1 (Shannon entropy), 1.5, and 2 (Collision entropy), resulting in five different preprocessed datasets on EDRan data. Each EDRan dataset contains event distribution randomness of “Team A” and “Team B” across 10 time periods ($t_{i};1\leq i\leq 10$), quantified using Rényi entropy with varying *α* values, along with the game result. Each preprocessed dataset contains one row per game included. Game result column is a binary value where 1 represents “Team A” win and 0 represents a “Team B” win.

### 2.4 Temporal analysis of EDRan with match winning performances

Next, Renyi entropy *α* values for which the EDRan was most associated with match winning results were evaluated.

First, EDRan difference between winners’ EDRan and losers’ EDRan for each time period was computed to evaluate whether winning teams prioritize unpredictability across all regions of the field vs dominant regions for match winning performances.

Subsequently, a correlation analysis between EDRan (for 0≤α≤2) and match outcomes was conducted. In an earlier work, match-winner classifiers were trained on a reduced feature set obtained by representing the EDRan difference between opposing teams as a single variable [28]. Same dimensionality-reduction technique was employed in this study to reduce the number of features from 20 features per game to 10 features per game.

Event distribution randomness (EDRan) difference between two teams for time period *t*_*i*_:

$$\Delta H_{\alpha }(t_{i})=\frac{H_{\alpha }^{A}(t_{i})-H_{\alpha }^{B}(t_{i})}{\delta t},$$ (5)

Here, $\Delta H_{\alpha }(t_{i})$ is EDRan difference between two teams for time period *i* (*t*_*i*_;1≤i≤10) where, EDRan computed with Renyi entropy alpha value *α*, $H_{\alpha }^{A}(t_{i})$ is EDRan of Team A for time period *t*_*i*_, $H_{\alpha }^{B}(t_{i})$ is EDRan of Team B for time period *t*_*i*_, δt is Duration of the time period.

EDRan difference between two teams has been normalized by the duration of time period as the duration of a time period varies from one half to another due to injury time.

This dimensionality reduction resulted in 10 temporal features per game, representing the differences in event distribution randomness (EDRan) between the two teams across ten time intervals. Separate datasets were created for each of the six considered *α* values (0, 0.1, 0.5, 1, 1.5, and 2) using the corresponding preprocessed EDRan datasets. Each EDRan difference dataset contained 10 features representing the EDRan differences between “Team A” and “Team B” over the 10 time intervals, along with a binary game outcome label, where 1 indicates a win for “Team A” and 0 indicates a win for “Team B”. A positive EDRan difference suggests that “Team A” maintained higher EDRan than “Team B” during the respective time period, while a negative difference indicates that “Team B” had greater EDRan than “Team A.” Therefore, a positive correlation between the EDRan difference and the match result label would indicate that EDRan is positively associated with match-winning performances, whereas a negative correlation would indicate that EDRan is negatively associated with match-winning performances.

### 2.5 Machine learning model development

Match-winner classification models were also developed using $\Delta H_{\alpha }(t_{i})$ features computed at α∈{0,0.1,0.5,1,1.5,2} to assess whether unpredictability concentrated in dominant regions or distributed across the entire field is more predictive of victory.

For the match winner classification machine learning model development, Random Forest (RF) classifier was considered due to its ability to handle a high number of features while maintaining robust performance with relatively small datasets, its robustness to nonlinearity and ease of interpretation via feature importances. Although SVM, XGBoost, and neural networks were initially considered, Random Forest was chosen for its interpretability through feature importance, as well as its computational efficiency and reduced need for extensive hyperparameter tuning compared to XGBoost and neural networks.

Its built-in feature importance evaluation allows for an assessment of each metrics contribution to match-winning performances. The Random Forest model is an ensemble learning method that constructs multiple decision trees and combines their outputs to improve predictive accuracy and reduce over-fitting. Additionally, random forest models provides more flexibility in hyperparameter tuning (e.g., number of trees, maximum depth), enabling performance optimization and fair comparison across varying *α* values.

The match winner classification model was evaluated using multiple performance metrics, including accuracy, F1-score, precision, recall, and Matthews Correlation Coefficient (MCC). To ensure a robust and comprehensive evaluation, each model’s average performance was estimated via repeated 50 rounds of 5-fold cross-validation. For each of 50 repetitions, the dataset was randomly shuffled and partitioned into five folds with random state of repetition number (1→50); in turn, four folds were used to train the model (80% train data) and the remaining fold served as the test set (20% test data).This yielded 50×5=250 evaluations per model (developed for each *α* value) across different data subsets.

Hyper-parameter tuning of the Random Forest model was performed using a grid search (GridSearchCV, scoring = accuracy) over $n_{\text{estimators}}\in \{50,100,200\}$, max_depth∈{None,10,20,30}, min_samples_split∈{2,5,10}, min_samples_leaf∈{1,2,4}, and bootstrap∈{True,False}; The random seed of the model was fixed at 42 for random forrest classifier. In order to avoid any hyperparameter data leakage, hyperparameter tuning was done to the training folds of each evaluation separately.

The mean, standard deviation of each performance metric were computed over these 250 evaluations. This repeated cross-validation approach provides a more reliable estimate of the model’s generalization capability by averaging performance over multiple training and testing splits.

To assess and compare changes in model performance relative to the baseline model using Shannon entropy (α→1), proposed in a previous published work [28], a t-test was conducted. This statistical test evaluates whether the means of two groups differ significantly. Specifically, model performances for *α* values of 0, 0.1, 0.5, 1.5, and 2 were each compared against the performance at α→1. In this context, a p-value less than 0.05 indicates a statistically significant difference between the two model performances. However, conducting multiple pairwise comparisons increases the risk of Type I error. To address this, both the Bonferroni correction and the Holm–Bonferroni correction were used.

## 3 Results

### 3.1 Temporal analysis results

**Fig 4:**
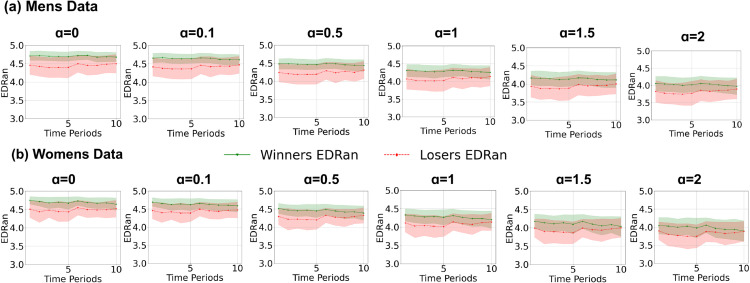
Temporal variations in mean Event Distribution Randomness (EDRan), computed using Rényi entropy for 0≤α≤2, across the ten time periods of the match for both men’s and women’s datasets. Standard deviations indicated in shaded area

The influence of EDRan on match outcomes was evaluated first. Initially, the variation of EDRan across the 10 time periods was analyzed separately for men’s and women’s games. Fig 4 illustrates the temporal trends in mean EDRan for winners and losers in both datasets. Overall, winners consistently demonstrated higher EDRan values compared to losers. However, the gap between the two groups tended to narrow as *α* increased.

**Fig 5:**
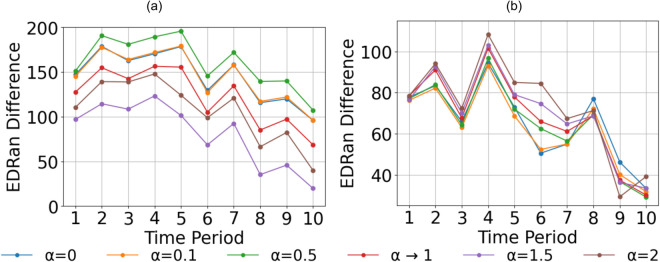
Cumulative EDRan difference between winners and losers across 10 time periods with (a) men’s dataset (b) women’s dataset.

To further investigate how this difference between winners and losers evolves over time, the cumulative EDRan difference (Winners’ EDRan – Losers’ EDRan) was calculated across all time periods for all the games in two datasets. Fig 5 displays the aggregated EDRan differences between winners and losers over the 10 time intervals for all matches in the dataset.

It was observed that cumulative EDRan difference between winners and losers is a positive value For both mens and womens data across 10 time periods. However, towards the last phases of the game EDRan difference has decreased. The greatest EDRan difference between winners and losers was observed at α=0.5, whereas for the women’s data, the maximum difference occurred at α=2. Although differences in EDRan between winners and losers are suggestive, they are insufficient on their own to robustly identify the optimal value of *α* most strongly associated with match outcomes.

**Fig 6:**
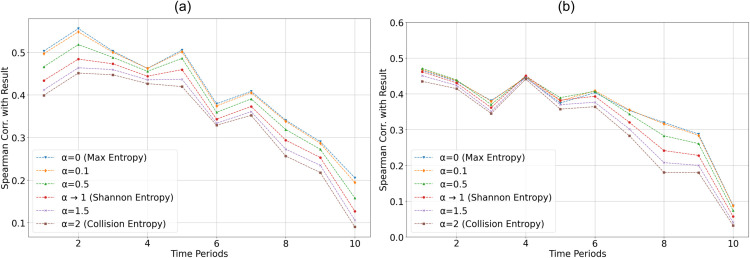
Spearman correlation between $\Delta H_{\alpha }(t_{i})$ (EDRan difference between “Team A” and “Team B”) quantified using Rényi entropy with 0≤α≤2 for ten $t_{i}$ time periods and game result (“Team A” win/ “Team A” loss) with (a) Men’s data (b) Women’s data.

The machine learning models of this work is trained using EDRan difference between two teams; “Team A” and “Team B” ($\Delta H_{\alpha }(t_{i})$) (quantified using Rényi entropy for 0≤α≤2) as features. Therefore, correlation between $\Delta H_{\alpha }(t_{i})$ and match winning results were also evaluated across ten *t*_*i*_ time periods. Given the non-normality characteristics (tested with Shapiro-Wilk test) of the distributions, Spearman correlation was employed. Fig 6 illustrates the Spearman correlation between event distribution randomness difference over ten *t*_*i*_ time periods and match-winning performances with men’s football (Fig 6(a)) and women’s football (Fig 6(b)).

Fig 6 shows that, the correlation between the $\Delta H_{\alpha }(t_{i})$ and match outcome reaches its highest value at maximum entropy (α=0) for the men’s dataset, and at α=0.1 for the women’s dataset. The mean Spearman correlation across all ten time periods tends to increase as α decreases. Across all considered values of α, the $\Delta H_{\alpha }(t_{i})$ is positively correlated with the match result, suggesting that higher EDRan values are associated with winning performances. However, the correlation coefficients decline toward the end of the game.

### 3.2 Match winner prediction model results

In existing literature, Shannon entropy (i.e., Rényi entropy as α→1) has commonly been used as the standard metric for quantifying event distribution randomness (EDRan). However, as illustrated in Fig 6, the correlation between $\Delta H_{\alpha }(t_{i})$ and match outcomes tends to decrease as α increases, for both men’s and women’s datasets. This raises the question of whether Shannon entropy is the optimal measure of randomness, as the results suggest that improvement in EDRan when α<1 is more strongly associated with match-winning performances than when α=1. However, this only capture the monotonic association between $\Delta H_{\alpha }(t_{i})$ and match result for each period; this does not necessarily indicate predictive strength. The predictive power may also depend on the extent to which EDRan differs between winners and losers across varying *α* values, offering insights into the distinct playing styles of both groups.

Therefore, to assess the predictive ability of EDRan difference across α values (0, 0.1, 0.5, 1, 1.5, and 2), match winner prediction models were developed using a simple Random Forest classifier. Tables 1 and 2 presents a comparison of the performance between models trained with Rényi entropy for *α* values 0,0.1,0.5,1,1.5,and 2 with men’s and women’s datasets respectively.

**Table 1:** Performance of match winner prediction model of men’s games at different *α* Values (mean ± std and 95% confidence interval). *p*-values correspond to accuracy vs. accuracy when α→0

*α*	Accuracy	Acc 95% CI	Precision	Prec 95% CI	Recall	Recall 95% CI	F1-score	F1 95% CI	MCC	MCC 95% CI
0	0.8061±0.0340	[0.8019,0.8103]	0.8243±0.0414	[0.8191,0.8295]	0.8364±0.0467	[0.8306,0.8422]	0.8292±0.0326	[0.8251,0.8333]	0.6050±0.0692	[0.5964,0.6136]
0.1	0.7984±0.0315	[0.7945,0.8023]	0.8222±0.0413	[0.8171,0.8273]	0.8223±0.0451	[0.8167,0.8279]	0.8211±0.0302	[0.8173,0.8249]	0.5905±0.0646	[0.5825,0.5985]
0.5	0.7810±0.0351	[0.7766,0.7854]	0.8014±0.0483	[0.7954,0.8074]	0.8156±0.0480	[0.8096,0.8216]	0.8071±0.0352	[0.8027,0.8115]	0.5546±0.0705	[0.5458,0.5634]
→1	0.7733±0.0348	[0.7690,0.7776]	0.7934±0.0500	[0.7872,0.7996]	0.8111±0.0455	[0.8054,0.8168]	0.8008±0.0349	[0.7965,0.8051]	0.5386±0.0697	[0.5299,0.5473]
1.5	0.7721±0.0349	[0.7678,0.7764]	0.7910±0.0484	[0.7850,0.7970]	0.8142±0.0469	[0.8084,0.8200]	0.8009±0.0324	[0.7969,0.8049]	0.5360±0.0720	[0.5270,0.5450]
2	0.7687±0.0341	[0.7645,0.7729]	0.7901±0.0482	[0.7841,0.7961]	0.8072±0.0480	[0.8012,0.8132]	0.7971±0.0339	[0.7929,0.8013]	0.5293±0.0690	[0.5207,0.5379]

**Table 2:** Performance of Match Winner Prediction Model of women’s*** games at different *α* values (mean ± std and 95% confidence interval) and *p*-values correspond to accuracy vs. accuracy when α→1.

*α*	Accuracy	Acc 95% CI	Precision	Prec 95% CI	Recall	Recall 95% CI	F1-score	F1 95% CI	MCC	MCC 95% CI
0	0.7530±0.0436	[0.7476, 0.7584]	0.7531±0.0627	[0.7453, 0.7609]	0.8236±0.0611	[0.8160, 0.8312]	0.7843±0.0439	[0.7790, 0.7896]	0.5009±0.0870	[0.4891, 0.5127]
0.1	0.7482±0.0499	[0.7421, 0.7543]	0.7497±0.0641	[0.7417, 0.7577]	0.8179±0.0650	[0.8098, 0.8260]	0.7799±0.0483	[0.7740, 0.7857]	0.4906±0.1028	[0.4770, 0.5042]
0.5	** 0.7560±0.0492 **	[0.7500, 0.7620]	** 0.7558±0.0664 **	[0.7476, 0.7640]	** 0.8289±0.0681 **	[0.8206, 0.8372]	** 0.7876±0.0467 **	[0.7818, 0.7934]	** 0.5096±0.0984 **	[0.4964, 0.5227]
→1	0.7512±0.0498	[0.7451, 0.7573]	0.7529±0.0684	[0.7446, 0.7612]	0.8195±0.0589	[0.8121, 0.8269]	0.7823±0.0481	[0.7765, 0.7881]	0.4966±0.1001	[0.4831, 0.5101]
1.5	0.7493±0.0436	[0.7439, 0.7547]	0.7553±0.0587	[0.7480, 0.7626]	0.8101±0.0610	[0.8026, 0.8176]	0.7793±0.0419	[0.7741, 0.7845]	0.4923±0.0895	[0.4803, 0.5043]
2	0.7465±0.0484	[0.7406, 0.7524]	0.7587±0.0679	[0.7505, 0.7670]	0.7970±0.0660	[0.7889, 0.8051]	0.7744±0.0466	[0.7686, 0.7801]	0.4888±0.0993	[0.4754, 0.5022]

***: The women’s dataset is underpowered; therefore, no definitive conclusions can be drawn.

To evaluate the statistical significance of performance improvements across varying *α* values, pairwise *t*-tests were conducted. Given the slight class imbalance in the dataset, the Matthews Correlation Coefficient (MCC) was selected as the evaluation metric, as it is relatively robust to data imbalance. However, performing multiple pairwise *t*-tests introduces the risk of inflating the family-wise Type I error. To control this Type I error, both Bonferroni and Holm–Bonferroni corrections were applied across the five planned comparisons against the α=1 baseline. Adjusted *p*-values were computed as $p_{adj}=\min(K\;p_{raw},1)$ with *K* = 5, and statistical significance was assessed at α=0.05 using $p_{adj}$. Under the Bonferroni correction, $p_{adj}$ corresponds to $p_{bonf}$, whereas under the Holm–Bonferroni correction, $p_{adj}$ corresponds to $p_{holm}$. This procedure mitigates inflation of false positives due to multiple testing. These t-test results with men’s and women’s data are provided in Table 3.

**Table 3:** Pairwise comparisons of MCC values against baseline α=1 for men’s and women’s data (reporting raw and adjusted *p*-values).

Gender	Comparison	Mean diff	*t*	df	$p_{\text{raw}}$	Hedges’ *g*	$p_{\text{bonf}}$	$p_{\text{holm}}$	Sig (Holm)
Men	α=0 vs α→1	0.0664	10.6893	497.97	3.78e-24	0.9546	1.89e-23	1.89e-23	True
	α=0.1 vs α→1	0.0519	8.6350	495.15	8.09e-17	0.7712	4.05e-16	3.24e-16	True
	α=0.5 vs α→1	0.0160	2.5518	497.94	1.10e-02	0.2279	5.51e-02	3.30e-02	True
	α=1.5 vs α→1	–0.0026	–0.4102	497.48	6.82e-01	–0.0366	1.00e+00	6.82e-01	False
	α=2 vs α→1	–0.0093	–1.4993	497.95	1.34e-01	–0.1339	6.72e-01	2.69e-01	False
Women***	α=0 vs α→1	0.0043	0.5126	488.51	6.08e-01	0.0458	1.00e+00	1.00e+00	False
	α=0.1 vs α→1	–0.0060	–0.6612	497.65	5.09e-01	–0.0590	1.00e+00	1.00e+00	False
	α=0.5 vs α→1	0.0130	1.4644	497.85	1.44e-01	0.1308	7.19e-01	7.19e-01	False
	α=1.5 vs α→1	–0.0043	–0.5063	491.89	6.13e-01	–0.0452	1.00e+00	1.00e+00	False
	α=2 vs α→1	–0.0078	–0.8747	497.97	3.82e-01	–0.0781	1.00e+00	1.00e+00	False

**MCC**: Matthews correlation coefficient.

**Comparison**: Pairwise comparison versus the baseline α→1 (Shannon).

**Mean diff**: Difference in mean MCC (*Δ*MCC = MCC_listed_− MCC_α→1_); positive favors the listed *α*.

**t**: Welch’s *t*-statistic from a two-sided test of zero mean difference.

**df**: Welch–Satterthwaite degrees of freedom.

$p_{\text{raw}}$: Unadjusted two-sided *p*-value.

**Hedges’ *g***: Bias-corrected standardized effect size; sign follows *Mean diff*.

$p_{\text{bonf}}$: Bonferroni-adjusted *p*-value across the set of pairwise comparisons.

$p_{\text{holm}}$: Holm–Bonferroni adjusted *p*-value across the same comparisons.

**Sig (Holm)**: Indicator for statistical significance at family-wise α=0.05 using Holm adjustment ($p_{\text{holm}}<0.05$).

***: The women’s dataset is underpowered; therefore, no definitive conclusions can be drawn.

With men’s data (Table 1), the model developed with Max entropy (α=0) outperformed the model based on Shannon entropy (α→1) across all evaluation metrics, including accuracy, F1-score, recall, precision, and MCC. This result was validated through a comprehensive evaluation involving fifty rounds of five-fold cross-validation, totaling 250 evaluations. These findings further reinforce the stronger correlation between $\Delta H_{\alpha }(t_{i})$ and match outcomes (Fig 6), suggesting that the correlation tends to decrease as the α value increases with men’s data.

Pairwise comparisons of MCC values against the baseline α=1 revealed that α=0 and α=0.1 achieved significantly higher MCC scores, with large effect sizes (Hedges’ *g* = 0.95 and 0.77, respectively; Holm-adjusted *p* < 10^−15^). A smaller but statistically significant improvement was also observed at α=0.5 (*g* = 0.23, Holm-adjusted *p* = 0.033), although the effect size was modest. In contrast, α=2 and α=1.5 did not differ significantly from the baseline (Holm-adjusted *p* > 0.2), indicating no performance advantage. These results indicate that lower *α* values, particularly α=0, provide the most robust improvements in MCC relative to the Shannon entropy baseline with men’s football data.

In contrast, the results for the women’s dataset ([tab:accuracies_womens]Table 2) did not exhibit a consistent pattern of decreasing predictive performance with increasing *α* values. The highest performance was observed at α=0.5, whereas the lowest occurred at α=2. Pairwise *t*-tests did not reveal any statistically significant differences in performance. It should be noted, however, that the women’s dataset is underpowered, and therefore no definitive conclusions can be drawn from these results.

**Fig 7:**
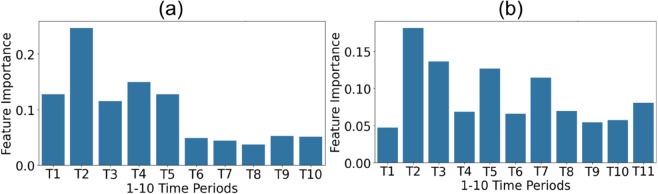
Random Forrest feature importance of the best performed models with (a) Mens data (α=0) (b) Womens data (α=0.5).

The best performing random forest models with men’s and women’s data (α=0 with men’s data and α=0.5 with women’s data) developed Random Forest model assigned greater importance to the early phases of the game, suggesting that these periods are more crucial in determining the match outcome. Fig 7 illustrates the feature importance of these Random Forest models across the ten time periods ($t_{i}:1\leq i\leq 10$).

## 4 Discussion

This study explores the most effective method for quantifying event distribution randomness (EDRan) in association football by comparing three approaches: emphasizing dominant or frequently used regions for ball movement, assigning equal weight to all used regions for ball movement, and using Shannon entropy, which weights each region based on its event distribution probability. To facilitate this comparison, Rényi entropy is employed with varying α values in the range 0≤α≤2. The α values at which the EDRan difference between two teams shows the highest correlation with match-winning outcomes are analyzed across ten defined time intervals (*t*_*i*_, where 1≤i≤10). Furthermore, to assess how predictive power varies with different α values, machine learning models are developed and evaluated using 50 rounds of five-fold cross-validation. Identifying the optimal α values that enhance predictive accuracy and correlation with match outcomes not only uncovers deeper insights into the game but also enables the development of a match winner classification model that outperforms existing models.

From Figs 4 and 5, it can be observed that winning teams generally maintain higher EDRan values compared to losing teams throughout the duration of the game. However, Fig 5 also shows that this difference in EDRan tends to diminish towards the later stages of the match. Additionally, as illustrated in Fig 6, the correlation between $\Delta H_{\alpha }(t_{i})$ and match outcomes decreases significantly in the final phases of the game for all *α* values. Furthermore, Random Forest models attribute greater importance to features from the early stages of the match, while assigning less importance to later time periods (Fig 7). Collectively, these findings may reflect the adoption of more cautious, risk-averse strategies by winning or leading teams towards the end of matches, a trend that aligns with observations in previous studies [17,18], where teams in the lead often prioritize defensive play to preserve their advantage towards the end of the game.

The correlation analysis between the Rényi entropy-based EDRan difference across teams ($\Delta H_{\alpha }(t_{i})$) and match-winning performance (Fig 6) revealed the strongest association at α=0 (maximum entropy) and the weakest at α=2 (collision entropy). These findings suggest that higher levels of spatial randomness, particularly involving the use of rare or less frequently occupied regions of the field, are generally associated with more favorable match outcomes. A likely explanation is that exploiting such regions increases unpredictability, disrupts defensive structure, and opens opportunities for sustained passing sequences or shots on goal. In contrast, when randomness is confined primarily to dominant regions (as captured at higher *α* values), defensive units are better able to anticipate and counter attacking movements, thereby limiting the tactical advantage.

With machine learning model performances, when α<1 with men’s data, models have shown statistically significant improvement compared to baseline model performance at α→1. When the Rényi entropy parameter *α* is low, the EDRan metric becomes more sensitive to rare or infrequently used spatial regions on the field. The observation that the highest model performance for men’s football data occurs at α=0 and significant improvements when α<1 suggests that winning men’s teams tend to utilize broader range of spatial regions, demonstrating greater event randomness across the entire field. Further analysis of EDRan differences between winning and losing teams (Fig 5) shows that, for men’s data, the greatest disparity also occurs at α=0, reinforcing the idea that winning teams tend to adopt a more spatially diverse and unpredictable playing style.

It is important to consider how this finding can be explained tactically and if it represents differences in physical performance. This pattern indicates men’s winning teams use a tactic of distributing play more evenly and unpredictably across the entire space in the field. Unpredictability that extends across all regions of the field may provide a greater tactical advantage, as it forces the opposition defense to account for a wider range of potential movements and makes anticipating attacking plays more difficult. Ultimately, this approach may facilitate the creation of open spaces by disrupting the opposition’s defense, enable safer or longer passing sequences, and generate opportunities for opportunistic shots on goal. In contrast, if randomness is restricted to dominant or frequently used regions, defenders can narrow their focus and adapt more easily to the limited set of possible actions, thereby reducing the effectiveness of the unpredictability. Additionally, a team’s ability to play unpredictably across a wider range of field zones than their opposition probably requires higher physical capacity. Higher physical capacity allows a team to move quickly into multiple field zones during an attacking phase, which would require the opposing team to perform more physical work in defense. If they are not trained for this level of physical performance they may experience higher levels of fatigue which could reduce the effectiveness of their defense, providing an advantage to the attacking team.

In contrast, the women’s data did not exhibit a consistent trend in model performance across varying *α* values. This may be attributable to the limited statistical power of the dataset or to other factors that differ from the men’s game. Consequently, although the results are presented, no definitive conclusions can be drawn. Nonetheless, the generalisability of the proposed approach, as well as potential differences in game-play randomness, could be more rigorously examined in future studies with sufficiently powered datasets.

In summary for top-tier men’s football data,

- Match-winning performance is positively associated with more unpredictable event distribution across the field, relative to concentrating unpredictability in a few dominant areas.
- Toward the end of matches, lower spatial randomness is observed for teams that won compared with earlier phases, whereas higher randomness is typically observed earlier in matches.

These patterns are associative rather than causal and may be influenced by potential confounding variables, including team and opponent strength, home/away status, game state/scoreline, and competition context.

The results from match winner prediction using machine learning models with men’s data showed statistically significant improvements for α<1 over the Shannon entropy-based baseline model α→1 Moreover, the top-performing model in this study not only surpassed the performance of the baseline model introduced in [28], which utilized EDRan differences between two teams calculated with Shannon entropy, but also outperformed comparable match outcome prediction models reported in recent literature. A summary comparison of the results from this work, the baseline model [28], and other related studies is presented in Table 4.

**Table 4:** Comparison of match winner prediction model results.

	Danisik et al. 2018 [21]	Almulla et al. 2023 [22]	Bandara et al. (2024) [28] Baseline Model Men’s	This work Men’s
Accuracy	70.213%	**80.77%**	79.95%	80.61%
F1-score		80.93%	81.89%	**82.92%**
Precision		80.34%	81.03%	**82.43%**
Recall		81.68%	83.02%	**83.64%**
MCC		**0.6167**		0.6050
Dataset	top five men’s club leagues from Europe	Qatar Stars men’s club league	top four men’s club leagues from Europe and Intl. competition	top four men’s club leagues from Europe and Intl competitions
Model	LSTM	GRU	GLM	RF
No. of features	138 featues on individual player perfromances	396 features from 22 player perf. eval. metrices	10 temporal features on EDRan	10 temporal features on EDRan

In comparison with existing studies, the present work adopts a smaller set of features and a simpler machine learning model, which enhances the interpretability of feature importance. Previous approaches [21,22] have primarily sought to maximize match-winner prediction accuracy using black-box recurrent neural networks, often at the expense of interpretability. In contrast, the focus here is on developing a temporal team performance evaluation metric. To this end, a single variable (EDRan) was extracted across 10 time steps, rather than the multiple features employed in prior studies, and a simple random forest model was implemented, enabling interpretation of feature importance with lower computational demands. Furthermore, while [21] incorporated historical data for model development, the current study relied exclusively on in-game data. Despite these simplifications, the approach achieved competitive predictive performance, underscoring the effectiveness of EDRan as a meaningful metric for evaluating team performance.

This study is subject to several limitations. First, the dataset is relatively small due to data availability constraints. While the men’s dataset was sufficiently powered, the women’s dataset was underpowered, preventing a reliable assessment of the approach in the women’s game. Second, the analysis was restricted to top-tier European competitions; therefore, the findings may not generalize to lower divisions, non-European leagues, or teams employing different tactical styles. Given that tactics evolve and can vary across skill levels, regions, age groups, and genders, the external validity of the findings beyond this cohort and period remains uncertain. Future research could expand the dataset to improve robustness, test generalizability across genders, and extend the analysis to different competitive levels, regions, and demographic cohorts to capture potential context-specific differences. Although standard cross-validation was used in this study, the authors acknowledge that temporal or group-wise cross-validation (e.g., by team or season) could further strengthen the assessment of model generalizability and reduce potential data leakage across similar match contexts. However, as the present analysis focuses not on team identity or season-level trends, but on the temporal sequence of in-game events to capture randomness and unpredictability in ball movement, standard cross-validation was deemed appropriate. Future work incorporating season-wise or team-based validation could provide additional insights into the stability of these findings over time and across teams. Moreover, this analysis did not incorporate game-state dependencies (e.g., leading, trailing, or tied situations), which are known to influence team behavior. This study was designed to examine overall temporal trends in event distribution randomness irrespective of current score or game context. However, future work could integrate game-state factors to provide deeper tactical insight into how teams adapt their strategies under different match conditions. Finally, drawn matches were excluded from the evaluation, as the primary aim was to identify factors contributing to winning performances. However, since draws are an integral part of football and may even be pursued strategically under certain circumstances, future work could incorporate drawn results to uncover additional patterns and strategic implications.

## 5 Conclusions

This study investigated the application of Rényi entropy with varying *α* values to quantify event distribution randomness (EDRan) in association football. The objective was to assess whether emphasizing randomness in ball movement confined to dominant regions of the field or distributing randomness more evenly across all regions better explains match outcomes. The analysis revealed that the strongest association between EDRan and match-winning performance occurred at α=0 (Max entropy), which reflects the breadth of distinct field regions utilized, regardless of frequency. This finding highlights the importance of maximizing spatial unpredictability, whereby successful teams exploit a wider range of field zones to disrupt opposition defenses and create advantageous opportunities.

Machine learning models developed with EDRan at α=0 for men’s data outperformed both the baseline model [28] and recently published match-winner classification models in the literature. These results underscore the potential of EDRan as a powerful performance evaluation metric and reinforce the tactical value of maintaining unpredictable ball movement across the entire field.

In conclusion, while football strategies may vary, with some teams favoring possession-based play and others focusing on defensive play with low-possession, successful teams tend to prioritize maintaining unpredictability across all regions of the field. These findings highlight the tactical advantage of fostering unpredictable event distributions across all regions of the field and suggest that spatial unpredictability in ball movement should be a key consideration in team strategy and performance evaluation.
